# The necessity of identifying the basal glucose set-point in the IVGTT for patients with Type 2 Diabetes

**DOI:** 10.1186/s12938-015-0015-7

**Published:** 2015-03-03

**Authors:** Nor Azlan Othman, Paul D Docherty, Jeremy D Krebs, Damon A Bell, J Geoffrey Chase

**Affiliations:** Department of Mechanical Engineering, Centre for Bio-Engineering, University of Canterbury, Private Bag 4800, Christchurch, 8054 New Zealand; Department of Medicine, University of Otago, Wellington, 6242 New Zealand; School of Medicine and Pharmacology Royal Perth Hospital Unit, The University of Western Australia, Perth, Western Australia 6009 Australia

**Keywords:** Basal glucose concentration, DISST, Insulin resistance, Insulin sensitivity

## Abstract

**Background:**

The model-based dynamic insulin sensitivity and secretion test (DISST) uses fasting glucose (*G*_*0*_) as the basal glucose (*G*_*B*_) concentration when assessing insulin sensitivity (*SI*). However, this model was developed in a healthy, normoglycaemic cohort. We sought to determine the suitability the DISST model has for individuals with established type 2 diabetes (T2D).

**Methods:**

14 participants with established T2D were recruited to take part in a dietary intervention study. Insulin-modified intravenous glucose tolerance tests (IM-IVGTT) were undertaken at week 0, 12 and 24 and were used with DISST model to identify *G*_*B*_. A total of 36 tests were conducted across 12 participants throughout the study. Measured *G*_*0*_ and identified *G*_*B*_ values were compared using a Kolmogorov-Smirnov (KS) and signed rank (RS) test for the cohort.

**Results:**

There were significant differences between the *G*_*0*_ and identified *G*_*B*_ values in this cohort (p_rs_ and p_ks_ < 0.0001), although both values were well correlated (*R* = 0.70). The residual plot demonstrates that the modified model captures the behaviour of the participants more accurately than the original model.

**Conclusions:**

This analysis has shown that *G*_*B*_ is an important variable for modelling the glycaemic behaviour in T2D. These findings suggest that the original DISST model, while appropriate for normoglycaemic cohorts, needs to model basal glucose level as a variable for assessing individuals with established T2D.

## Background

Type 2 diabetes mellitus (T2DM) is a metabolic disease that affects the body’s ability to regulate glucose concentrations [1-3]. T2DM is characterized by fasting and postprandial hyperglycaemia [4] and causes comorbidities with significant personal and economic cost [5]. The hyperglycaemia is attributed to a combination of impaired insulin utilization (insulin resistance) and a limited ability to compensate with insulin production (net insulin deficiency).

The ability to quantify both insulin sensitivity and insulin secretion is essential to improving the understanding of the complex physiology underlying type 2 diabetes. Mathematical models of glycaemic dynamics have been coupled with clinical data to identify key aspects in the pathogenesis of type 2 diabetes. The dynamic insulin sensitivity and secretion test (DISST) incorporates a clinical protocol similar to the insulin-modified intravenous glucose tolerance test (IM-IVGTT) [6,7] and measures the participant glucose, insulin and C-peptide responses. The DISST data modelling and data fitting methods were customized to the clinical protocol and allow a robust measurement of insulin sensitivity (*SI*) that avoids the problems encountered with IVGTT assessment in insulin resistant patients [8-11]. The DISST *SI* value is highly correlated to the euglycaemic hyperinsulinaemic clamp (EIC) *SI* value (*R* = 0.81), which is widely regarded as the reference method [12].

However, the DISST model uses the participant’s measured fasting glucose concentration (*G*_*0*_) as their basal glucose concentration (*G*_*B*_). The *G*_*B*_ term in the DISST model effectively has the role of determining the set-point towards which the modelled glucose response tends, where this choice matches assumptions in all other model-based tests [6,9,13-15]. However, the DISST model was developed in a relatively healthy, normoglycemic cohort [16,17]. In contrast, studies show that *G*_*0*_ levels and insulin concentrations are slightly higher in the morning than their overnight “basal” levels, especially for participants with diabetes [18-21]. Therefore, the evidence suggests that *G*_*B*_ and *G*_*0*_ should be treated as separate entities for individuals with established diabetes as the levels are determined by relative insufficiencies in *SI,* endogenous insulin secretion (*U*_*N*_) and rates of gluconeogenesis [22-24].

We sought to determine whether a novel modelling approach that identifies *G*_*B*_ as a variable in individuals with established type 2 diabetes more accurately reflects glucose dynamics.

## Methods

### Participants

Fourteen individuals with established type 2 diabetes mellitus were recruited from the Wellington region of New Zealand to take part in an Atkins-Based low carbohydrate dietary intervention study. Recruited participants were aged between 30 and 65 with a BMI range of 34 to 46 kg · m^−2^ at baseline. Participants were excluded if they had major physiological or psychological illness at the time of testing. Pregnant or lactating females were also excluded. Two participants discontinued the intervention, the first citing personal reasons, and the second left the study due to a renal stone. Twelve participants each underwent three IM-IVGTT over a 24 week period resulted in a total of 36 tests for the study. Participants had their age and BMI recorded (median [IQR]; 47.5 [42.5, 54.5] and 40.40 [37.48, 43.48], respectively). Full demographic details and results of the intervention study have been previously described [25]. Ethics approval for this study was provided by the New Zealand Ministry of Health, Central Regional Ethics Committee.

### Clinical procedure

The clinical protocol utilised in this study was similar to the protocol defined by Ward *et al.* [7]. A 0.2 g · kg^−1^ glucose bolus was administered at *t* = 1 minute and then an infusion of insulin that was intended to replicate the insulinaemic response of a normoglycaemic individual was administered. An insulin infusion was started at *t* = 2 minutes at a rate of 3.5 mU · kg^−1^ · min^−1^ and was reduced to 0.5 mU · kg^−1^ · min^−1^ at *t* = 7 minutes. Further reductions occurred at *t* = 17 minutes, to 0.25 mU · kg^−1^ · min^−1^, and at *t* = 50 minutes, to 0.1 mU · kg^−1^ · min^−1^. The infusion of mU · kg^−1^ · min^−1^ was maintained for the remainder of the procedure. Venous blood samples were taken into fluoride oxalate tubes at times: *t* = −10, −5, −1, 0, 2, 3, 4, 5, 6, 8, 10, 12.5, 15, 20, 25, 30, 35, 40, 50, 60, 70, 80, 90, 100, 120, 140, 160, 180, 210, 240, 270 and 300 minutes. Blood samples were assayed for glucose and insulin concentration using standard commercial assays at an accredited laboratory.

### Model

#### Dynamic Insulin Sensitivity and Secretion Test (DISST) Model

This analysis used the DISST models of interstitial insulin kinetics and glucose dynamics [16,17]:

1 $$ \dot{Q}=\frac{n_I}{V_Q}I-\left({n}_C+\frac{n_I}{V_Q}\right)Q $$

2 $$ \dot{G}=-{p}_G\left(G-{G}_B\right)-SI\left(GQ-{G}_B{Q}_B\right)+\frac{P_X}{V_G} $$

where equation nomenclature is shown in Table 1.

**Table 1:** **Nomenclature of the DISST model**

**Variable**	**Unit**	**Description**	**Role**
*I*	mU · L^−1^	Plasma insulin concentration	Measured
*G*	mmol · L^−1^	Plasma glucose concentration	Measured
*G*_*B*_	mmol · L^−1^	Basal plasma glucose concentration	Measured
*Q*	mU · L^−1^	Interstitial insulin concentration	Simulated
*Q*_*B*_	mU · L^−1^	Basal interstitial insulin concentration	Simulated
*V*_*Q*_	L	Interstitial insulin distribution volume	*a-priori*
*n*_*I*_	L · min^−1^	Plasma-interstitial diffusion rate	*a-priori*
*n*_*C*_	min^−1^	Interstitial insulin degradation rate	*a-priori*
*P*_*X*_	mmol · min^−1^	Exogenous glucose input rate	*a-priori*
*p*_*G*_	min^−1^	Non-insulin mediated glucose disposal rate	*a-priori*
*V*_*G*_	L	Glucose distribution volume	Identified
*SI*	L · mU^−1^ · min^−1^	Insulin sensitivity	Identified

*V*_*Q*_, *n*_*I*_ and *n*_*C*_ are defined a-priori based on anatomical functions [17,26-28] while DISST model sets *p*_*G*_ as a constant at 0.004 min^−1^ [17].

#### Parameter identification

Interstitial insulin (*Q*) was simulated via integrating factors and a linear interpolation of *I*.

1a $$ Q={e}^{-{\displaystyle {\int}_0^t{n}_C+\frac{n_I}{V_Q}dt}}\left({Q}_0+{{\displaystyle {\int}_0^te}}^{{\displaystyle {\int}_0^t{n}_C+\frac{n_I}{V_Q}dt}}\frac{n_I}{V_Q}Idt\right) $$

where *Q*_*0*_ is determined assuming a steady state at *t* = −10 minutes:

3 $$ {Q}_0=\frac{\frac{n_I}{V_Q}{I}_0}{n_C+\frac{n_I}{V_Q}} $$

The DISST model typically sets *G*_*B*_ as equal to *G*_*0*_ [16,17]. Hence, *G*_*0*_ acts as a surrogate basal glucose concentration level. However, for individuals with elevated fasting glucose, this assumption may not be accurate [18-21], and should be tested.

In this analysis, *G*_*B*_ was identified in concert with *SI* and *V*_*G*_. The typical approach used with the DISST model identifies only *SI* and *V*_*G*_. Thus, the outcomes of the three-parameter (*G*_*B*_, *SI*, *V*_*G*_) model were compared to the outputs of the typical two-parameter model (*SI*, *V*_*G*_).

A Gauss Newton parameter identification method was used to identify the participant-specific models, with objective function defined:

4 $$ {\mathrm{x}}_{i+1}={\mathrm{x}}_i-\alpha {\left({\mathrm{J}}^{\mathrm{T}}\mathrm{J}\right)}^{-1}{\mathrm{J}}^{\mathrm{T}}\uppsi $$

where x_*i*_ = [*G*_*Bi*_, *SI*_*i*_, *V*_*Gi*_]^*T*^ and *i* is the iteration number. The Jacobian matrix (**J**) and the residual matrix (**ψ**) are defined:

$$ \mathrm{J}\left({\mathrm{x}}_i\right)=\left[\begin{array}{ccc}\hfill \frac{\delta {\psi}_1}{\delta {G}_{Bi}}\hfill & \hfill \frac{\delta {\psi}_1}{\delta S{I}_i}\hfill & \hfill \frac{\delta {\psi}_1}{\delta {V}_{Gi}}\hfill \\ {}\hfill \frac{\delta {\psi}_2}{\delta {G}_{Bi}}\hfill & \hfill \frac{\delta {\psi}_2}{\delta S{I}_i}\hfill & \hfill \frac{\delta {\psi}_2}{\delta {V}_{Gi}}\hfill \\ {}\hfill \vdots \hfill & \hfill \vdots \hfill & \hfill \vdots \hfill \\ {}\hfill \frac{\delta {\psi}_n}{\delta {G}_{Bi}}\hfill & \hfill \frac{\delta {\psi}_n}{S\delta {I}_i}\hfill & \hfill \frac{\delta {\psi}_n}{\delta {V}_{Gi}}\hfill \end{array}\right],\kern1.12em \uppsi \left({\mathrm{x}}_i\right)=\left[\begin{array}{c}\hfill G\left({x}_i,{t}_1\right)-{G}_S\left({t}_1\right)\hfill \\ {}\hfill G\left({x}_i,{t}_2\right)-{G}_S\left({t}_2\right)\hfill \\ {}\hfill \vdots \hfill \\ {}\hfill G\left({x}_i,{t}_n\right)-{G}_S\left({t}_n\right)\hfill \end{array}\right] $$

where *n* is the number of measured samples, *G*(**x**_*i*_*,t*_*1*_) is the modelled glucose concentration at *t* = *t*_*1*_ given **x**_i_, *G*_*S*_(*t*_*1*_) is the measured glucose level at *t* = *t*_*1*_.

The Jacobian was numerically evaluated using perturbations of [*δG*_*B*_, *δSI*, *δV*_*G*_] = [10^− 3^, 10^8^, 10^− 3^]. These perturbation values were 0.1% of the order of magnitude of the expected parameter values. Glucose samples between *t* = 1 and *t* = 10 minutes were disregarded by the identification methods, as this period is heavily influenced by mixing kinetics that are not captured by the whole body model of glucose metabolism [17,29].

Identifying *G*_*B*_ in concert with *SI* and *V*_*G*_ can cause identified parameter trade off in some cases [8]. The value of *V*_*G*_ was thus limited to physiologically measured bounds from other studies [16,17,30,31]. In particular, *V*_*G*_ was limited to the range of [0.12*Bw*, 0.25*Bw*] where bodyweight (*Bw*) is measured in kg and the coefficients have units of l · kg^−1^, which is a standard estimation approach linking volume to an easily measured value. Similarly, *G*_*B*_ was limited to a minimum of 3 mmol · L^−1^.

### Analysis

Model residuals and interpretation of population trends were used to assess the performance of the *G*_*B*_ identified - DISST model. The *p*-values are defined with signed ranksum (p_rs_) and Kolmogrov Smirnov test (p_ks_) to assess median and variability. All analysis was undertaken using MATLAB (R2013b, Mathworks, Inc., Natick, MA, USA).

## Results

**Figure 1:**
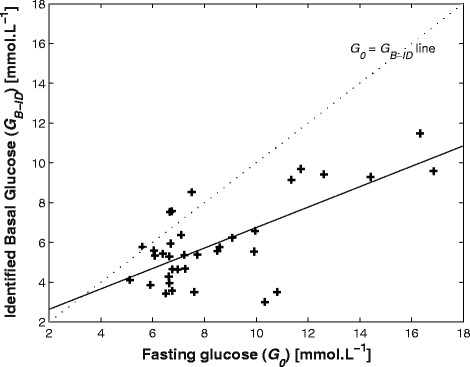
**Relationship between fasting glucose (** ***G***_***0***_ **) and identified basal glucose (** ***G***_***B-ID***_ **) across tests.** The 1:1 *G*_*0*_ = *G*_*B-ID*_ line (dots) is to show the bias between approaches. The solid line has *R* = 0.70.

**Figure 2:**
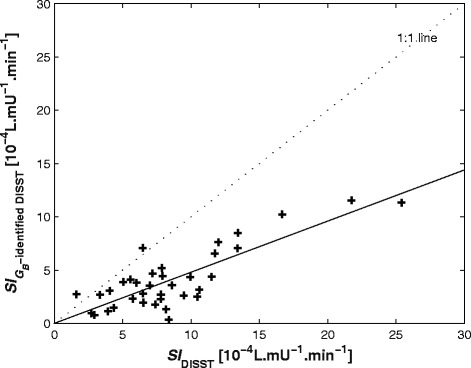
**Relationships between** ***SI*** **values (based on DISST and** ***G***_***B***_ **-identified DISST model) across tests.** The 1:1 line (dots) is to show the bias between approaches. The solid line has *R* = 0.83.

Figures 1 and 2 shows the individual relationships between fasting glucose, (*G*_*0*_), identified basal glucose (*G*_*B-ID*_) and identified *SI* from normal DISST and *G*_*B*_-identified DISST model across all participants and tests. Note the bias about the 1:1 line indicating that on average, the identified, model-based basal set point for glucose was significantly lower than the fasting rate for this cohort with diabetes. Figure 1 shows there were significant differences between the *G*_*0*_ and *G*_*B-ID*_ values in this cohort (Signed-ranksum: p_rs_ < 0.0001, Kolmogorov Smirnov: p_ks_ < 0.0001). In general, *G*_*0*_ was higher than the *G*_*B-ID*_ value, with only 4 exceptions over 36 results (11.1%). Although there was a significant difference in the levels of *G*_*B-ID*_ and *G*_*0*_, they were relatively well correlated (*R* = 0.70), indicating moderately consistent bias in the relationship between values.

Figure 2 shows the effect that identifying *G*_*B-ID*_ has on identified insulin sensitivity values. There is a reasonably strong correlation between the *SI* values between normal DISST and *G*_*B*_-identified DISST model (*R* = 0.83). The bias indicates that by identifying basal glucose, the model captures consistently lower *SI* values for those with established T2DM.

**Figure 3:**
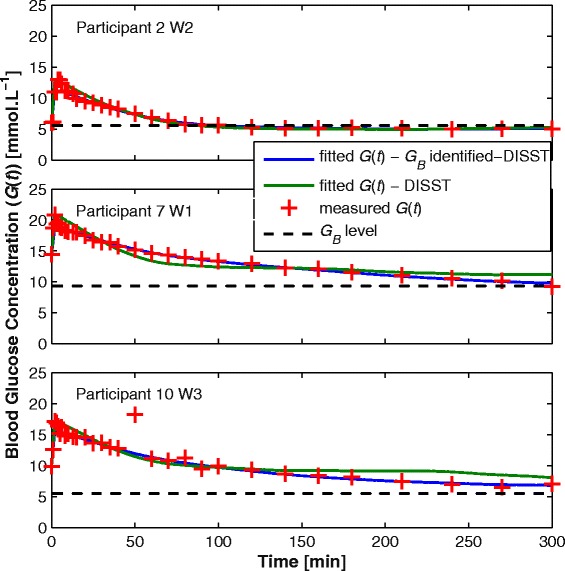
**Blood glucose participant-specific profile (** ***G*** **(** ***t*** **)) for participants 2, 7, 10 with normal DISST and modified DISST identifying** ***G***_***B***_ **.**

**Figure 4:**
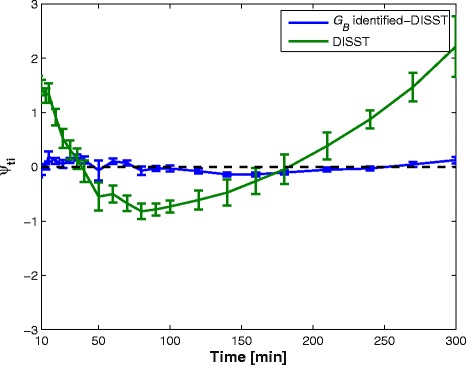
**Residual error (mean and standard error, SE = SD/√N) between the measured glucose data and the response model by Equation** **2** **.** The residuals reflect model accuracy after bolus dosing and mixing errors are passed.

Figure 3 shows the fitted glucose profiles and measured glucose data from 3 different participants. It also shows that the identified *G*_*B-ID*_ levels are well below than *G*_*0*_ as depicted in Figure 1. Figure 4 illustrates the residual errors of both the typical DISST model and the proposed three parameter identified model that identifies basal glucose. Note that the glucose samples taken within 10 minutes of glucose injection were ignored due to un-modelled mixing effects.

## Discussion

This is the first study to demonstrate that glucose excursions are more accurately modelled using basal glucose as the variable in the DISST model, rather than fasting glucose for individuals with established type 2 diabetes. The typical approach employed when using the DISST model defines the fasting glucose (*G*_*0*_) as the basal glucose concentration (*G*_*B*_). Hence, the glucose response defined by the original model typically tends towards the measured basal value. However, this analysis has shown that this assumption is not valid for a cohort with established diabetes. This discrepancy in the assumption is evidenced by the significant distinction in the values of *G*_*0*_ and *G*_*B*_ that reduces as the participant glycaemic control improved across the time points of the dietary intervention study [25]. Figure 1 shows that while most participants had elevated fasting glucose levels, the identified basal level was often much closer to a lower value seen in healthy subjects. In particular, 14 of the 36 *G*_*B*_ values identified were in the normal reference range of 4–5.6 mmol∙L^−1^ [32] while only two of 36 *G*_*0*_ values were in that range.

However, there were some participants for whom *G*_*B-ID*_ remained very high throughout the intervention. Of the three participants that exhibited *G*_*B-ID*_ values greater than 9 mmol · L^−1^, two were first diagnosed 10 years prior to the trial. In contrast, the mean duration of diabetes for the whole cohort was 4.4 years (SD = 1.0 year). This outcome indicates a possible mechanism of dysfunction in type 2 diabetes that develops during the course of the disease, and matches growing dysfunction over time in these individuals. However, this study lacks the numbers required for conclusive proof of this trend.

Figure 2 shows the effects of insulin sensitivity (*SI*) values have from identified *G*_*B*_ values are significantly lower than *G*_*0*_ values particularly for these type 2 diabetes participants. Hypothetically, if models to set *G*_*B*_ to be equal to *G*_*0*_, *SI* will account for low glucose level rather than *G*_*B*_ and is thus modelled as a higher *SI* value. A recent study shows that the type 2 diabetes subjects have *SI* values in the magnitude of 2–4 × 10^−4^ L · mU^−1^ · min^−1^ [16]. Although, there is not enough evidence to prove the agreeable range of *SI* value for type 2 diabetes participants, it is understandable that lower *SI* value contributes to the pathogenesis of type 2 diabetes as *SI* is inversely proportional to insulin resistance (*IR*) [3].

Figure 3 shows the blood glucose profiles of three participants as modelled by the typical two-parameter (*SI*, *V*_*G*_) and three-parameter (*G*_*B*_, *SI*, *V*_*G*_) DISST model. While the typical two-parameter DISST model fails to fully capture the responses of the participants, the amended three-parameter model captures the behaviours more closely. This outcome is confirmed by the residual plots in Figure 4 that indicate a much smaller, yet consistent trend about the measured data. This change implies the modified model captures previously un-modelled effects or poor a-priori estimates in the interstitial insulin kinetic model.

The original DISST model was developed [17] and validated [12] in relatively normoglucose tolerant cohorts. In these cohorts, the incidence of impaired fasting glucose was relatively low, and thus, the assumption of *G*_*0*_ equals to *G*_*B*_ was well founded. However, the glycaemic behaviour of the cohort used in this analysis showed that this assumption was most likely to be invalid. In particular, the lower glucose levels achieved in the later part of the test would be falsely attributed to insulin sensitivity rather than a *G*_*B*_ value that was lower than *G*_*0*_. The significantly biased residuals showed in Figure 4 show that the typical DISST model cannot capture all dynamics of this cohort without identifying *G*_*B*_ directly.

Overall, these results indicate that the original two-parameter approach, while appropriate for normoglycaemic cohorts [16,17], is less suitable for individuals with established type 2 diabetes. Furthermore, it is most likely that the outcomes of this study would be applicable to pre-diabetic individuals that have elevated blood glucose. However, this assertion remains to be determined.

Neither DISST approach accurately captures the peak value of the measured blood glucose data. This particular result was due to the disregarded glucose data within 10 minutes of glucose injection. This data was rejected due to the unmodelled effects of intravascular mixing [29]. A second compartment to model local/global mixing kinetics could have been added. However, this addition was deemed unnecessary, as such compartments do not add value to the DISST modelled outcomes [17]. The approach used was intended to avoid over-fitting to mixing effects or fitting the simple DISST model of global glucose dynamics to the local glucose mixing data.

Although, this analysis was done in a small cohort, the outcomes are significant as it has shown that *G*_*B*_ is an important variable when modelling the glycaemic behaviour in type 2 diabetes. It also showed that *G*_*B*_ can be quite different to the typically assumed *G*_*0*_ value, and that it may also have some diagnostic value. These findings suggest that the *G*_*B*_ value should be treated as a variable in DISST model for this cohort. Further validation in a much larger cohort will provide a solid foundation for these findings.

## Conclusions

This analysis has shown the presence of a dysfunction in the basal (set-point) glucose in individuals with type 2 diabetes. The magnitude of the dysfunction has been shown to be linked to insulin sensitivity and the degree of fasting glucose. This analysis suggests that the basal glucose is a more appropriate variable for individuals with type 2 diabetes, as using the fasting glucose measurement as the basal set-point was shown to be a poor assumption for this cohort - although this requires confirmation in a larger study with a clamp as the reference.

## Abbreviations

.: Physiological conditions
T2DM: Type 2 diabetes mellitus
.: Insulin sensitivity tests
EIC: Euglycaemic hyperinsulinaemic clamp
IVGTT: Intra-venous glucose tolerance tests
IM-IVGTT: Insulin-modified IVGTT
DISST: Dynamic insulin sensitivity and secretion test
.: Blood assays
*G*_*0*_: Fasting glucose
*G*_*B*_: Basal glucose
*G*_*B-ID*_: Identified basal glucose
.: Common model parameters
*SI*: Insulin sensitivity (L · mU^−1^ · min^−1^)
*U*_*N*_: endogenous insulin secretion (mU · min^−1^)
*I*: Plasma insulin concentration (mU · L^−1^)
*G*: Plasma glucose concentration (mmol · L^−1^)
*G*_*B*_: Basal plasma glucose concentration (mmol · L^−1^)
*Q*: Interstitial insulin concentration (mU · L^−1^)
*Q*_*B*_: Basal interstitial insulin concentration (mU · L^−1^)
*V*_*Q*_: Interstitial insulin distribution volume (L)
*n*_*I*_: Plasma-interstitial diffusion rate (L · min^−1^)
*n*_*C*_: Interstitial insulin degradation rate (min^−1^)
*P*_*X*_: Exogenous glucose input rate (mmol · min^−1^)
*p*_*G*_: Non-insulin mediated glucose disposal rate (min^−1^)
*V*_*G*_: Glucose distribution volume (L)
